# Knowledge, attitudes, and practices of caregivers of functionally disabled older adults regarding nutritional management

**DOI:** 10.3389/fnut.2025.1660965

**Published:** 2025-11-05

**Authors:** Jing Jia, Ying Guo, Li Tian, Wen Li, Xianyan Cao, Yanping Shang

**Affiliations:** Department of Cardiopulmonary Rehabilitation, Guangdong Work Injury Rehabilitation Hospital, Guangzhou, China

**Keywords:** caregivers, functionally disabled persons, nutritional management, knowledge, attitudes, practice, cross-sectional study

## Abstract

**Objective:**

This study aimed to assess the knowledge, attitudes, and practices (KAP) of caregivers of functionally disabled older adults regarding nutritional management.

**Methods:**

A multicenter cross-sectional survey was conducted on caregivers of functionally disabled older adults between April and June 2025 across Guangdong, Guangxi, and Hunan Province, utilizing an online questionnaire to collect demographic information and evaluate knowledge and practice scores. In this study, caregiver burden measured by the Zarit Burden Interview Short Form (ZBI-12) was defined as the attitudinal construct within the KAP framework. Structural equation model (SEM) was constructed to evaluate the interrelationships among the KAP.

**Results:**

A total of 550 valid responses were included, yielding an effective response rate of 86.21%. The majority of respondents were female (71.09%). The mean knowledge, attitude, and practice scores were 15.30 ± 6.23 (possible range: 0–24), 18.53 ± 10.01 (possible range: 0–48), and 33.44 ± 7.01 (possible range: 9–45), respectively. SEM indicated significant associations from knowledge to attitude (*β* = −0.478, *p* = 0.015) and practice (*β* = 0.589, *p* = 0.018), and from attitude to practice (*β* = −0.286, *p* = 0.011). In addition, knowledge showed an indirect association with practice through attitude (*β* = 0.137, *p* = 0.007).

**Conclusion:**

Caregivers of functionally disabled older adults demonstrated limited knowledge, moderate attitudes, and proactive practices in nutritional management. Intervention strategies should prioritize targeted educational support that enhances caregivers’ nutritional knowledge while simultaneously addressing attitudinal barriers and caregiver burden to optimize caregiving behaviors.

## 1 Introduction

The accelerating global aging process has transformed the health management of functionally disabled older populations into one of the most pressing public health challenges of the 21st century. According to projections from the United Nations, by 2050, the number of adults aged 65 or over will be twice as big as the amount of children under the age of five and also surpass the number of adolescents aged between 15 and 24 years, with improvements in survival expected to add approximately 5 years to the life expectancy at birth for the world’s population (1). In China, this demographic transition is equally pronounced, with 264 million people aged over 60 years and 190 million people aged over 65 years recorded in 2020 (2). Among the global elderly population, disability and chronic disease are closely linked to advancing age, with substantial numbers of functionally impaired older people requiring comprehensive care support (3).

Malnutrition represents a particularly critical concern in this population, with prevalence rates varying significantly across care settings, from 3% in community environments to approximately 30% in rehabilitation and subacute care facilities (4). Functionally disabled older adults are particularly vulnerable to malnutrition due to multiple factors including swallowing difficulties, reduced appetite, medication side effects, and physical limitations that impair food preparation and consumption (5). The consequences are severe, with malnourished disabled older adults experiencing a 2.5-fold increased risk of mortality, 40% higher rates of infection complications, and significantly prolonged hospital stays compared to well-nourished counterparts (6).

The Knowledge, Attitudes, and Practices (KAP) framework serves as a fundamental diagnostic research tool that illuminates a population’s comprehension, beliefs, and behaviors regarding specific health topics, operating on the premise that knowledge positively influences attitudes, which subsequently shape behavioral practices (7). In the context of functionally disabled older adults, family caregivers play a crucial role in implementing nutritional management interventions. This is particularly significant in China, where the unique “9,073” elderly care model indicates that approximately 90% of elderly individuals live at home, 7% depend on community support, and only 3% reside in professional institutions (8). The complexity of this relationship becomes evident when considering that caregivers face multifaceted challenges, including physical harm, psychological pressure, and responsibility management burdens (9). Research has demonstrated that caregivers of moderately and severely functionally disabled older adults show significantly higher caregiving burden scores and poorer health-related quality of life compared to those caring for individuals with mild disabilities (8). Understanding caregivers’ KAP profiles is therefore crucial for developing targeted interventions that can improve both caregiver competence and care outcomes. The KAP framework has been widely used to examine health-related behaviors across diverse populations because of its intuitive logic linking knowledge, attitudes, and practices. Nevertheless, its application to caregiver populations requires nuance, since attitudes in caregiving are often operationalized not as favorable or unfavorable dispositions but as perceived burden or role appraisal. This may lead to atypical patterns, where greater knowledge can increase awareness of caregiving challenges, thereby elevating perceived burden, while burden in turn may constrain the translation of knowledge into practice. These dynamics suggest that while the KAP model provides a useful heuristic, the interpretation of associations should be contextualized within caregiving research.

However, limited research has specifically examined KAP regarding nutritional management among caregivers of functionally disabled older adults, with existing studies primarily focusing on general caregiving competence or specific disease conditions rather than comprehensive nutritional care practices (10, 11). In the present study, we interpreted attitude within the KAP model as caregiver burden, consistent with studies that conceptualize emotional strain and role appraisal as an attitudinal component of caregiving. Therefore, this study aimed to assess the knowledge, attitudes, and practices (KAP) of caregivers of functionally disabled older adults regarding nutritional management.

## 2 Materials and methods

### 2.1 Study design and participants

This cross-sectional survey was conducted on caregivers of functionally disabled older adults between April and June 2025 across Guangdong, Guangxi, and Hunan Province. This study has been approved by the Ethics Committee of the Guangdong Work Injury Rehabilitation Hospital for biomedical research (Approval No.: AF/SC-07/2025.07) and has obtained informed consent from the research participants. The inclusion criteria were as follows: 1. primary caregivers of disabled older adults; 2. aged 18 years or older; and 3. voluntary participation in the study. The exclusion criteria included: 1. caregivers whose care recipients were not disabled due to aging but due to acute or temporary conditions (e.g., short-term recovery from fractures or trauma) with an expected return to self-care in the near future; 2. caregivers who were only partially involved in caregiving and not responsible for diet- or nutrition-related tasks; and 3. caregivers currently participating in other interventional studies that may affect the nutritional status or caregiving practices of disabled older adults.

### 2.2 Questionnaire

The questionnaire was developed based on the previous studies and relevant guidelines, such as *Recommendations for home nutrition therapy for disabled patients in China* (12) and *Expert consensus on nutrition diagnosis and treatment in elderly patients* (13). Following the initial draft, the instrument was revised in response to expert review by a panel of five specialists, each with over 20 years of clinical experience in fields such as internal medicine nursing, geriatric nursing, or rehabilitation nursing, providing in-depth evaluations and suggestions to improve the content validity and clarity of the questionnaire. Experts suggested that the questionnaire requires improvements in content comprehensiveness, clarity of expression, and overall professionalism. Specifically, they recommended incorporating additional common geriatric conditions (e.g., malignancies, dementia), refining item wording to enhance accuracy and reduce ambiguity, removing irrelevant or leading questions, including items on community-based elderly care resources, and further specifying behavior-related items to improve their operability and measurement precision. A pilot test involving 43 participants was then conducted to assess its reliability. The questionnaire showed an overall Cronbach’s *α* coefficient of 0.968, suggesting strong internal consistency. To assess the construct validity of the self-designed KAP questionnaire, a Confirmatory Factor Analysis (CFA) was conducted. The results indicated that the questionnaire exhibited good structural validity, with all factor loadings above the recommended threshold and no significant cross-loadings. Detailed fit indices and factor loadings are provided in Supplementary Table S1 and Supplementary Figure S1, supporting the suitability of the questionnaire for subsequent analyses.

The final version of the questionnaire consisted of four sections: demographic characteristics, knowledge, attitudes, and practices. The knowledge domain included 12 items, scored as 2 for “very familiar,” 1 for “somewhat familiar,” and 0 for “unfamiliar,” yielding a total score range of 0–24. The attitudinal component was represented by caregiver burden, assessed using the 12-item Zarit Burden Interview Short Form (ZBI-12), a validated tool widely used to evaluate the emotional, social, and physical strain perceived by caregivers. This approach follows prior work that considers burden as an evaluative dimension of caregivers’ attitudes toward their role. The ZBI-12 evaluates emotional, social, and physical strain experienced in caregiving, with each item scored from 0 (“never”) to 4 (“nearly always”), yielding a total score range of 0–48; higher scores indicate greater perceived burden (14). The total score was categorized into two levels of caregiver burden: scores from 0 to 9 indicated no to mild burden, while scores of 10 and above indicated moderate to high burden (15). The practice section contained 9 items, each rated on a five-point Likert scale ranging from 5 (“always”) to 1 (“never”), with total scores ranging from 9 to 45. For analytical purposes, scores exceeding 70% of the maximum in each domain were considered indicative of adequate knowledge, and proactive caregiving practices (16).

### 2.3 Questionnaire distribution and quality control

Convenience sampling was employed to recruit participants from 16 hospitals across Guangdong, Guangxi, and Hunan provinces. Of these, 13 hospitals consented to participate in the study, yielding a response rate of 81.25%. A detailed list of participating hospitals and departments is provided in Supplementary Table S2. Data were collected through both online electronic questionnaires and offline paper-based questionnaires. Questionnaires were distributed in both outpatient and inpatient departments of the participating units. A team of five trained research assistants was involved in the data collection process. One assistant was responsible for participant eligibility screening, three for administering the questionnaires, and one for checking data quality. During on-site survey administration, research assistants were available to address participants’ questions, ensuring accurate comprehension of questionnaire items. For the electronic survey, the questionnaire was developed using the Wenjuanxing platform,^1^ and a QR code was generated for distribution via WeChat. To maintain data integrity, each IP address was allowed only one submission, and all items were set as mandatory. The research team conducted manual reviews of all submitted questionnaires to ensure completeness, logical consistency, and response reliability.

### 2.4 Sample size

The sample size was determined based on a rule of thumb for structural equation modeling, which recommends a minimum of 10–15 participants per observed variable (17). Given that the questionnaire included 33 observed variables (12 for knowledge, 12 for attitude, and 9 for practice), the minimum required sample size was estimated to be between 330 and 495. Considering possible non-responses and invalid submissions, we aimed to recruit at least 600 participants.

### 2.5 Statistical analysis

Descriptive analyses were performed to summarize the demographic characteristics of the participants and the distribution of knowledge (K), attitude (A), and practice (P) scores. Continuous variables were expressed as means and standard deviations (SD), whereas categorical variables and individual item-level responses were reported as frequencies and percentages. Group differences in KAP scores across sociodemographic variables were assessed using univariate analyses for independent samples. For comparisons involving two groups, the Mann–Whitney U test was applied, while the Kruskal–Wallis H test was used for comparisons among three or more groups. These non-parametric methods were chosen because the data did not meet the assumptions of normality or homogeneity of variance, as assessed by the Shapiro–Wilk and Levene’ s tests. Spearman’ s rank correlation coefficient was calculated to evaluate the monotonic associations among knowledge, attitude (caregiver burden), and practice scores, given the ordinal nature of the data and their non-normal distribution. Missing data were minimal because all items in the electronic questionnaire were set as mandatory, and paper-based responses were checked for completeness by research assistants; thus, no imputation procedures were required. As multiple univariate comparisons were conducted, results were interpreted with caution, but no formal adjustment for multiple testing was applied in order to retain sensitivity to potential associations. To further examine the interrelationships between K, A, and P, a structural equation model (SEM) was constructed using the individual items from each dimension as observed indicators. The model was evaluated using standard fit indices, including the root mean square error of approximation (RMSEA), incremental fit index (IFI), Tucker–Lewis index (TLI), and comparative fit index (CFI). Parameter estimates were reported in standardized form. Model estimation and modification were based on theoretical considerations and empirical fit indicators. All statistical analyses were conducted using IBM SPSS Statistics for Windows, version 27.0 (IBM Corp., Armonk, NY, United States), and AMOS version 26.0 (IBM Corp., Armonk, NY, United States). A two-tailed *p*-value less than 0.05 was considered to indicate statistical significance.

## 3 Results

Initially, a total of 638 questionnaires were initially collected. After excluding 3 questionnaires from participants who did not consent to data usage, 1 questionnaire from a respondent under 18 years old, 5 questionnaires identified as outliers, 19 questionnaires where the functionally disabled older adults were under 60 years old, and 60 questionnaires with incorrect answers to trap questions, 550 valid questionnaires remained for final analysis, with an effective rate of 86.21%.

### 3.1 Demographic information on participants

This study included 550 caregivers of functionally disabled older adults, predominantly female (71.09%), with a mean age of 44.55 ± 12.46 years. Caregivers were primarily immediate family members (46%) or professional caregivers (36.18%), caring for elderly individuals with a mean age of 71.90 ± 7.99 years (Table 1). A series of chronic diseases were the main cause of disability among the functionally disabled older adults (411 cases), followed by aging of body functions and decline of senses (253 cases) (Supplementary Figure S2A). When it comes to the current chronic conditions of the functionally disabled older adults, the highest proportion is cardiovascular diseases (349 cases), followed by metabolic diseases (190 cases) and neurological diseases (178 cases) (Supplementary Figure S2B).

**Table 1:** Demographic characteristics and KAP scores.

Characteristics	N (%)	Knowledge, mean ± SD	*P*	Attitude, mean ± SD	*P*	Practice, mean ± SD	*P*
	550 (100)	15.30 ± 6.23		18.53 ± 10.01		33.44 ± 7.01	
Age of respondent (years)	44.55 ± 12.46						
Age of the functionally disabled older person (years)	71.90 ± 7.99						
Gender of respondent			0.055		0.811		0.921
Male	159 (28.91)	14.52 ± 6.25		18.18 ± 9.98		33.45 ± 7.54	
Female	391 (71.09)	15.62 ± 6.21		18.67 ± 10.02		33.44 ± 6.80	
Gender of the functionally disabled older person			0.118		0.195		0.160
Male	299 (54.36)	15.75 ± 6.26		18.07 ± 10.58		33.86 ± 7.09	
Female	251 (45.64)	14.77 ± 6.17		19.07 ± 9.27		32.94 ± 6.90	
Household monthly income (Yuan)			<0.001		<0.001		<0.001
<5,000	200 (36.36)	16.36 ± 6.23		15.87 ± 9.66		34.49 ± 7.40	
5,000–10,000	214 (38.91)	15.69 ± 6.02		17.95 ± 9.76		33.89 ± 6.71	
10,000–20,000	105 (19.09)	13.73 ± 6.35		24.64 ± 9.51		31.74 ± 6.87	
>20,000	31 (5.64)	11.13 ± 4.65		18.97 ± 6.44		29.39 ± 4.24	
Relationship with the functionally disabled older person			<0.001		<0.001		0.001
Immediate family member	253 (46.00)	13.59 ± 5.88		20.58 ± 10.17		32.10 ± 6.94	
Non-immediate family member	55 (10.00)	15.07 ± 6.23		18.09 ± 8.64		34.15 ± 7.03	
Professional caregiver	199 (36.18)	17.40 ± 6.08		16.49 ± 9.84		34.82 ± 6.82	
Volunteer or staff from other rehabilitation institutions	43 (7.82)	15.98 ± 6.12		16.44 ± 9.29		34.07 ± 7.10	
Caregiving experience	3.83 ± 3.83						
Received relevant training			<0.001		<0.001		<0.001
Yes	254 (46.18)	18.13 ± 5.83		15.96 ± 9.82		35.51 ± 6.89	
No	296 (53.82)	12.88 ± 5.51		20.73 ± 9.64		31.67 ± 6.63	
Health condition of the functionally disabled older person			0.023		<0.001		0.006
Excellent	67 (12.18)	14.66 ± 6.19		16.16 ± 9.20		33.28 ± 7.43	
Good	88 (16.00)	16.24 ± 5.96		15.42 ± 9.72		34.98 ± 6.96	
Fair	256 (46.55)	15.64 ± 6.38		18.45 ± 10.13		33.89 ± 6.83	
Poor	99 (18.00)	14.96 ± 6.32		21.76 ± 9.93		31.94 ± 7.14	
Very poor	40 (7.27)	13.00 ± 5.22		21.83 ± 8.19		31.18 ± 6.36	
Special dietary needs			0.045		0.008		0.751
Yes	319 (58.00)	15.79 ± 6.56		19.42 ± 10.52		33.55 ± 7.03	
No	231 (42.00)	14.63 ± 5.70		17.29 ± 9.13		33.30 ± 7.01	
Disability level*			0.007		0.011		0.590
Mild disability	147 (26.73)	14.94 ± 6.20		16.45 ± 9.72		33.46 ± 6.94	
Moderate disability	204 (37.09)	14.45 ± 5.97		19.74 ± 9.85		33.05 ± 6.61	
Severe disability	199 (36.18)	16.45 ± 6.38		18.82 ± 10.18		33.82 ± 7.47	
Feeding method			0.325		0.054		0.008
Oral feeding	368 (66.91)	15.40 ± 6.35		18.01 ± 10.38		33.96 ± 7.16	
Nasogastric feeding	182 (33.09)	15.12 ± 6.00		19.57 ± 9.14		32.39 ± 6.61	
Pressure ulcers			0.666		0.909		0.219
Yes	74 (13.45)	15.03 ± 5.96		17.93 ± 10.48		32.65 ± 7.73	
No	476 (86.55)	15.35 ± 6.28		18.62 ± 9.94		33.57 ± 6.90	

*According to internationally accepted standards, six indicators—eating, dressing, getting in and out of bed, using the toilet, walking indoors, and bathing—are used to assess the degree of disability. If one to two items are “unable to perform,” the individual is classified as having *mild disability*; three to four items as *moderate disability*; and five to six items as *severe disability*. Therefore, in the questionnaire, this item was designed as follows: “Among the six basic activities (eating, dressing, getting in and out of bed, using the toilet, walking indoors, bathing), how many is the functionally disabled older person unable to perform?”. Values are presented as mean ± SD or n (%). K, knowledge; A, attitude (caregiver burden); P, practice. Group comparisons were conducted using Mann–Whitney U test or Kruskal–Wallis H test as appropriate.

Lower household monthly income was significantly associated with higher knowledge but lower attitude scores (both *p* < 0.001). Professional caregivers had significantly better knowledge and practice outcomes than immediate family members (*p* < 0.001). Training was associated with significantly higher knowledge, more positive attitudes, and better practices (all *p* < 0.001). Caregivers of elderly individuals with severe disabilities showed higher knowledge scores compared to those caring for moderately disabled individuals (*p* = 0.007), though practices did not differ significantly. Better health status of the care recipient was associated with more positive attitudes and better practices among caregivers (*p* < 0.001). Caregivers managing nasogastric feeding reported significantly lower practice scores compared to those managing oral feeding (*p* = 0.008) (Table 1). After correcting for multiple comparisons, the main findings were summarized in Supplementary Table S3.

### 3.2 Knowledge, burden, and practice domain scores

The mean scores for the three domains were 15.30 ± 6.23 for knowledge (possible range: 0–24), 18.53 ± 10.01 for attitude (caregiver burden) (possible range: 0–48), and 33.44 ± 7.01 for practice (possible range: 9–45). The distribution of knowledge dimensions showed that the three questions with the highest number of participants choosing the “Unclear” option were ‘For functionally disabled older adults receiving nasogastric feeding, the food temperature should be moderate, generally between 38 and 40 °C, to avoid irritating the gastric mucosa with overly hot or cold food.’ (K6) with 24.36%, “During nasogastric feeding, the elderly individual should be in a semi-upright position or with the head elevated by at least 30–45° to reduce the risk of aspiration.” (K7) with 20.91%, and “After meals, bedridden elderly individuals should have the upper body elevated by 30–45° for about 30 min to prevent food reflux.” (K8) with 20.55%. Responses to the attitude dimension showed that 16.36% always and 22.55% often feel that the patient depends on them (A3), 10.73% always and 20.18% often feel that they do not have enough time for themselves because of caregiving (A6), 9.64% always and 18.18% often feel that caregiving takes up too much of their time (A1). When it comes to the importance of nutritional care for the functionally disabled older adults in all aspects of caring, 57% think it is very important, 29% think it is important and the rest were neutral or think it is not important (Supplementary Figure S3). Responses to the practice dimension showed that 19.82% rarely and 11.82% never read up on the latest information about elderly nutrition management to improve their knowledge (P9), 12.91% rarely and 7.64% never track the elderly individual’s weight changes and adjust the diet accordingly (P6), 11.09% rarely and 4.36% never prepare a nutritionally balanced diet for the functionally disabled older individual every day (P1) (Tables 2–4).

**Table 2:** Distribution of responses to knowledge items.

Knowledge items, n (%)	Very familiar	Heard of it	Not clear
1. Compared with healthy elderly individuals, functionally disabled older adults generally require higher intake of protein and calories to maintain bodily functions and promote recovery.	209 (38.00)	254 (46.18)	87 (15.82)
2. functionally disabled older adults need to pay special attention to the intake of vitamin D, calcium, iron, and B vitamins to prevent osteoporosis, anemia, and neurological dysfunction.	210 (38.18)	247 (44.91)	93 (16.91)
3. functionally disabled older adults may have poor digestive function and should consume easily digestible foods, such as soft foods, liquid diets, and high-fiber foods, to promote digestive health.	243 (44.18)	235 (42.73)	72 (13.09)
4. Consuming foods rich in dietary fiber, such as whole grains, legumes, nuts, and seeds, helps maintain intestinal health.	254 (46.18)	224 (40.73)	72 (13.09)
5. Diets should be adjusted based on the elderly individual’s specific health conditions; for example, individuals with diabetes or hypertension should follow corresponding dietary guidelines.	242 (44.00)	243 (44.18)	65 (11.82)
6. For functionally disabled older adults receiving nasogastric feeding, the food temperature should be moderate, generally between 38 and 40 °C, to avoid irritating the gastric mucosa with overly hot or cold food.	230 (41.82)	186 (33.82)	134 (24.36)
7. During nasogastric feeding, the elderly individual should be in a semi-upright position or with the head elevated by at least 30–45° to reduce the risk of aspiration.	256 (46.55)	179 (32.55)	115 (20.91)
8. After meals, bedridden elderly individuals should have the upper body elevated by 30° to 45° for about 30 min to prevent food reflux.	262 (47.64)	175 (31.82)	113 (20.55)
9. Avoid offering sticky or hard-to-chew foods, such as glutinous rice cakes or jelly, to elderly individuals with swallowing difficulties.	269 (48.91)	208 (37.82)	73 (13.27)
10. Record the elderly individual’s food intake and types of food to monitor their nutritional status and any potential changes in dietary preferences.	239 (43.45)	233 (42.36)	78 (14.18)
11. Adjust the diet according to seasonal changes; for example, provide light and easy-to-digest foods in summer and warm foods in winter to maintain body temperature.	239 (43.45)	228 (41.45)	83 (15.09)
12. Regardless of whether the elderly individual is fed orally or via nasogastric tube, the amount and type of food and the individual’s reaction should be recorded, and the diet should be reasonably adjusted based on medical advice.	233 (42.36)	233 (42.36)	84 (15.27)

Values are presented as n (%). K, knowledge.

**Table 3:** Distribution of responses to attitude items.

Attitude items, n (%)	Never	Occasionally	Sometimes	Often	Always
1. Do you feel that caregiving takes up too much of your time?	147 (26.73)	119 (21.64)	131 (23.82)	100 (18.18)	53 (9.64)
2. Do you feel stressed between caring for the patient and trying to meet other responsibilities such as work or household tasks?	154 (28.00)	121 (22.00)	127 (23.09)	101 (18.36)	47 (8.55)
3. Do you feel that the patient depends on you?	106 (19.27)	88 (16.00)	142 (25.82)	124 (22.55)	90 (16.36)
4. Do you feel nervous when the patient is around you?	290 (52.73)	96 (17.45)	85 (15.45)	59 (10.73)	20 (3.64)
5. Do you feel that your health has suffered because of your involvement in caring for the patient?	197 (35.82)	97 (17.64)	129 (23.45)	85 (15.45)	42 (7.64)
6. Do you feel that you do not have enough time for yourself because of caregiving?	146 (26.55)	109 (19.82)	125 (22.73)	111 (20.18)	59 (10.73)
7. Do you feel that your social life has suffered because of your involvement in caring for the patient?	166 (30.18)	106 (19.27)	126 (22.91)	104 (18.91)	48 (8.73)
8. Do you feel that you could do a better job in caring for the patient?	106 (19.27)	84 (15.27)	134 (24.36)	134 (24.36)	92 (16.73)
9. Do you feel that since taking on the caregiving role, living your own life as you want is no longer possible?	155 (28.18)	111 (20.18)	143 (26.00)	87 (15.82)	54 (9.82)
10. Do you wish someone else could take over the care of the patient?	243 (44.18)	100 (18.18)	109 (19.82)	66 (12.00)	32 (5.82)
11. Do you feel uncertain about what to do when it comes to caring for the patient?	189 (34.36)	111 (20.18)	148 (26.91)	61 (11.09)	41 (7.45)
12. Overall, how would you rate the burden of caregiving?	169 (30.73)	115 (20.91)	129 (23.45)	79 (14.36)	58 (10.55)

Values are presented as n (%). A, attitude (caregiver burden).

**Table 4:** Distribution of responses to practice items.

Practice items, n (%)	Always	Often	Sometimes	Seldom	Never
1. I prepare a nutritionally balanced diet for the functionally disabled older individual every day.	166 (30.18)	174 (31.64)	125 (22.73)	61 (11.09)	24 (4.36)
2. I adjust the diet plan according to the health condition of the functionally disabled older individual.	154 (28.00)	178 (32.36)	140 (25.45)	58 (10.55)	20 (3.64)
3. When the elderly individual refuses certain foods, I seek alternatives to ensure adequate nutrition.	164 (29.82)	167 (30.36)	143 (26.00)	57 (10.36)	19(3.45)
4. I ensure that the functionally disabled older individual drinks enough water every day.	224 (40.73)	162 (29.45)	102 (18.55)	44 (8.00)	18 (3.27)
5. I monitor the food intake of the functionally disabled older individual.	204 (37.09)	174 (31.64)	110 (20.00)	44 (8.00)	18 (3.27)
6. I always track the elderly individual’s weight changes and adjust the diet accordingly.	152 (27.64)	152 (27.64)	133 (24.18)	71 (12.91)	42 (7.64)
7. If the elderly individual has difficulty swallowing, I take appropriate measures, such as modifying food texture.	176 (32)	169 (30.73)	135 (24.55)	46 (8.36)	24 (4.36)
8. I ask about and record the elderly individual’s food preferences and aversions to optimize meal planning.	168 (30.55)	185 (33.64)	131 (23.82)	47 (8.55)	19 (3.45)
9. I read up on the latest information about elderly nutrition management to improve my knowledge.	145 (26.36)	111 (20.18)	120 (21.82)	109 (19.82)	65 (11.82)

Values are presented as n (%). P, practice.

### 3.3 Correlation analysis

Further correlation analysis revealed negative correlations between knowledge scores and attitude scores (*r* = −0.391, *p* < 0.001), as well as attitude scores and practice scores (*r* = −0.476, *p* < 0.001). Additionally, knowledge scores were positively correlated with practice scores (*r* = 0.642, *p* < 0.001) (Supplementary Table S4).

### 3.4 SEM analysis

**Figure 1:**
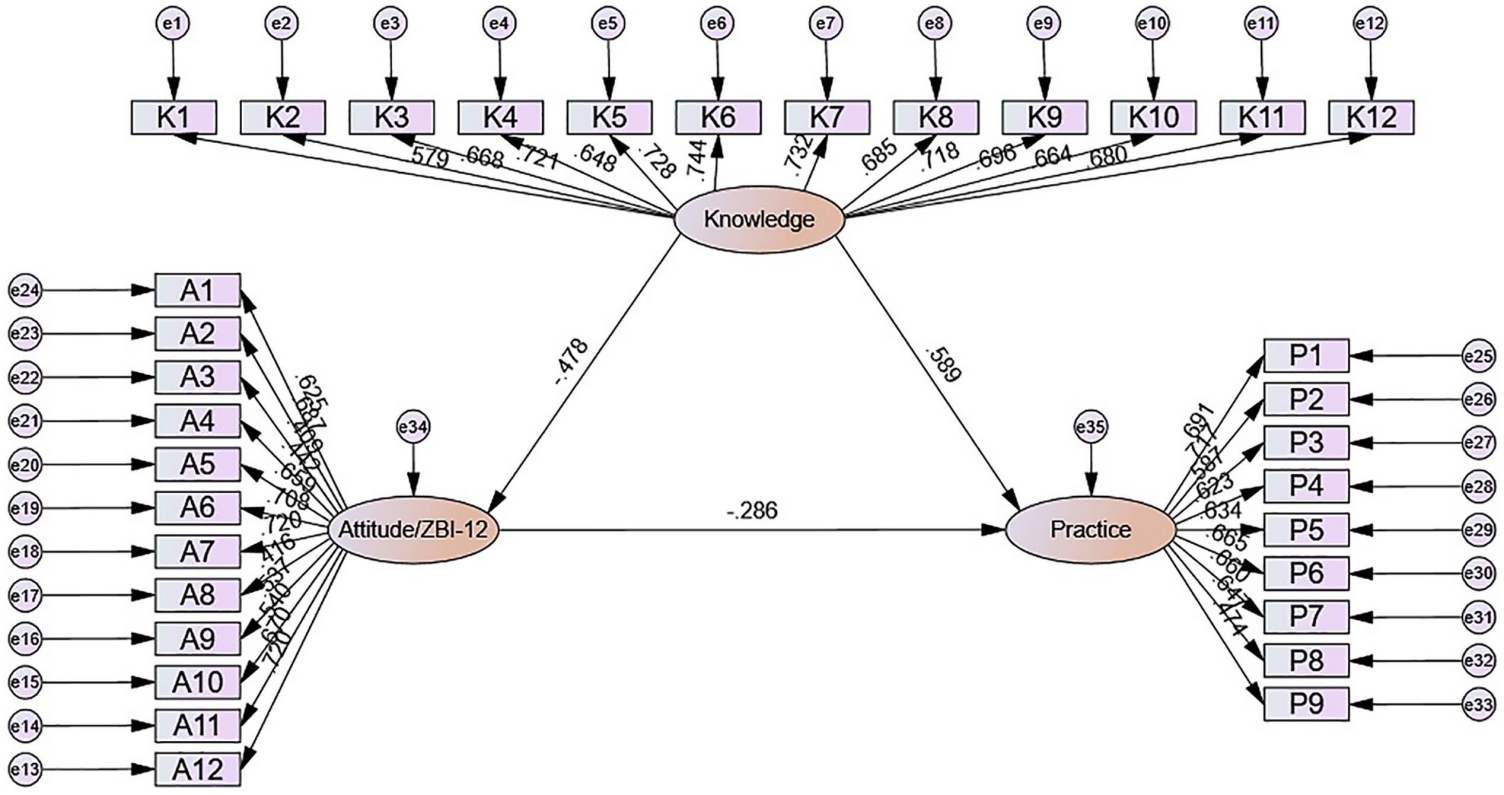
Path diagram of the structural equation model.

The SEM demonstrate a highly favorable model fit indices (CMIN/DF value: 3.564, RMSEA value: 0.068, IFI value: 0.845, TLI value: 0.833, and CFI value: 0.844), suggesting a well-fitting model (Supplementary Table S5). The mediation analysis indicated significant associations of knowledge with attitude (*β* = −0.478, *p* = 0.015) and practice (*β* = 0.589, *p* = 0.018), as well as an association between attitude and practice (*β* = −0.286, *p* = 0.011). Knowledge also showed an indirect association with practice through attitude (*β* = 0.137, *p* = 0.007) (Table 5 and Figure 1).

**Table 5:** Bootstrap to explore mediating associations of major pathways.

Model paths	Standardized Total effects		Standardized direct effects		Standardized indirect effects	
	*β* (95%CI)	*P*	*β* (95%CI)	*P*	*β* (95%CI)	*P*
Knowledge→Attitude	−0.478 (−0.531, −0.392)	0.015	−0.478 (−0.531, −0.392)	0.015		
Knowledge→Practice	0.725 (0.663, 0.773)	0.015	0.589 (0.512, 0.652)	0.018		
Attitude→Practice	−0.286 (−0.366, −0.219)	0.011	−0.286 (−0.366, −0.219)	0.011		
Knowledge→Practice					0.137 (0.105, 0.183)	0.007

Standardized coefficients (*β*) and 95% confidence intervals (CI) are reported.

## 4 Discussion

Caregivers demonstrated insufficient knowledge, moderately strained attitudes, yet generally proactive practices concerning the nutritional management of functionally disabled older adults. These findings underscore the need for targeted educational interventions to enhance caregivers’ nutritional knowledge, which may, in turn, optimize their attitudes and improve care practices in community and home-based elderly care settings.

This study explored the KAP of caregivers regarding nutritional management for functionally disabled older adults in China, revealing a set of interrelated but uneven patterns. While most caregivers acknowledged the importance of nutritional care, as indicated by the dominant portion identifying it as a critical aspect of caregiving, this recognition did not consistently translate into adequate knowledge or uniformly supportive attitudes. The gap between perceived importance and actual knowledge may reflect a broader issue of knowledge recognition without access to relevant, practical information, a phenomenon previously noted in similar caregiving populations. For example, in a study on family caregivers of patients with cerebral infarction in China, the mean knowledge score was 6.67 ± 1.73, while attitudes and practices were relatively higher at 32.95 ± 2.46 and 28.64 ± 4.39, respectively, with strong positive effects observed from knowledge to both attitudes and practices (10). In contrast, our study revealed a higher knowledge range (0–24) but comparatively moderate knowledge levels and negatively correlated attitudes, highlighting contextual and content-specific challenges in elderly nutritional care. Similarly, Shahin and Hussien (2021) reported that caregivers of children with epilepsy initially exhibited low knowledge, attitude, and practice scores, which significantly improved after a structured educational intervention. Their findings underscore that without systematic and targeted support, caregivers tend to struggle in translating recognition into actionable practice, reinforcing our observation of a mismatch between perceived importance and operational knowledge (18).

The SEM findings offer additional insight into these discrepancies. Knowledge showed a strong association with caregiving practice, both directly and indirectly through attitude. However, the negative association between knowledge and attitude suggests a more complex dynamic. As caregivers acquire more nutritional knowledge, they may become increasingly aware of the limitations in their caregiving environments or personal capacity, which can, in turn, increase perceived stress or emotional burden. This finding indicates that the traditional KAP pathway may not fully capture the complexity of caregiving contexts. Alternative frameworks, such as stress-appraisal-coping models or role theory, may also help explain why greater knowledge is linked to heightened burden and why burden may constrain the application of knowledge in practice. Alternative explanations should also be considered. On the one hand, this discrepancy is explained by the fact that attitude was measured using the Zarit Burden Interview Short Form (ZBI-12), which reflects caregiver burden rather than a positive attitudinal construct. Accordingly, higher knowledge may help reduce burden, while greater burden may hinder good practices, thereby yielding negative correlations. Another possibility relates to sample characteristics, as participants were primarily recruited from hospital settings and may not represent the full spectrum of caregiving experiences. Contextual influences, such as limited community support and the predominance of family-based care in China, may also exacerbate the sense of burden even among more knowledgeable caregivers. Taken together, these findings suggest that the KAP framework, while informative, should be interpreted flexibly in caregiving research, with recognition of burden as a negative attitudinal construct. This interpretation aligns with prior observations in caregiving research, particularly in settings where structural support is limited and the caregiving role is assumed with minimal preparation or training (19, 20). However, given the cross-sectional design of our study, these findings should be interpreted as associations rather than causal effects. It should also be acknowledged that the use of the ZBI-12 to represent the attitudinal component is a conceptual limitation, as this instrument measures caregiver burden rather than attitudes specifically related to nutritional management.

Attitudinal responses indeed reflected considerable emotional strain. A substantial proportion of caregivers reported feeling overwhelmed by the demands of caregiving, with concerns extending to time constraints, social withdrawal, and uncertainty in task execution. These expressions are consistent with patterns documented in other healthcare systems, where caregivers of individuals with high dependency levels often experience a tension between duty and capacity, particularly in the absence of external resources or shared responsibilities (11, 19). It is worth noting that these burdens were not uniformly distributed. For example, caregivers of individuals in poorer health conditions tended to report more negative attitudes.

In contrast, caregiving practices were generally proactive and stable. Many respondents indicated consistent engagement in dietary planning, hydration monitoring, and adaptation of food texture based on swallowing ability. These practices demonstrated strong positive correlations with knowledge scores and inverse associations with burdened attitudes, reinforcing the pathway identified in the structural equation model. However, areas requiring more specialized or adaptive responses—such as nasogastric feeding and tracking weight changes—were less frequently implemented. This pattern suggests that while caregivers may perform well on routine tasks, the application of more nuanced or technical strategies remains limited. Such findings mirror reports from comparable caregiving settings where practice quality improves with knowledge exposure but plateaus when caregivers lack ongoing support or advanced guidance (11, 21).

Socioeconomic factors further stratified these outcomes. Caregivers with prior training consistently demonstrated higher scores across all three dimensions, indicating the efficacy of even limited structured instruction. The association between lower income and higher knowledge and practice scores, although initially counterintuitive, may reflect more hands-on caregiving involvement among households unable to afford formal care services. Conversely, wealthier respondents may delegate daily care, resulting in limited personal familiarity with nutrition management protocols. These findings highlight how household context influences not only caregiving responsibilities but also the accumulation of caregiving competencies (22, 23).

Most caregivers reported managing elderly individuals with multiple chronic conditions—primarily cardiovascular and metabolic diseases—followed by mental health issues and physical injuries. These health challenges often require specific dietary interventions and close monitoring, yet such conditions were not matched by correspondingly high knowledge scores in the relevant areas. Table 2 reveals persistent uncertainty around topics such as micronutrient needs, seasonal diet adjustments, and safety precautions during feeding, particularly for nasogastric procedures. Inconsistent familiarity with these aspects points to structural weaknesses in information dissemination and caregiver preparation, a limitation previously observed in non-institutional elder care environments (23, 24).

Emotional burden, as captured in the attitudinal dimension, appeared to be shaped by the absence of institutional scaffolding. Caregivers frequently reported wishing to relinquish their responsibilities or feeling that their own lives had been significantly disrupted. Although such sentiments are not uncommon in long-term care contexts, the degree to which they co-occur with knowledge gaps and weak support systems warrants greater policy attention. These findings echo broader regional challenges in elder care where informal caregivers carry disproportionate responsibilities without sufficient access to training or psychosocial support (22, 25).

Our findings indicate that increasing caregiver knowledge alone may inadvertently elevate perceived burden. Therefore, a more systematic training infrastructure is urgently needed, integrating both procedural knowledge and strategies to mitigate stress. Caregiver education programs should focus not only on general nutritional principles but also on procedural knowledge specific to clinical conditions and feeding modalities. Training should be delivered through accessible formats, such as short video modules or community workshops, and adapted to varied literacy levels. Content should cover areas identified in this study as especially weak, including safe feeding techniques, the physiological impact of common chronic diseases, and signs of malnutrition. Evidence from related intervention studies suggests that even brief but targeted educational input can lead to marked improvements in caregiving outcomes, provided the material is practically oriented and culturally appropriate (26, 27).

Alongside technical training, the emotional dimensions of caregiving require greater institutional acknowledgment. Strategies to mitigate caregiver stress could include peer support networks facilitated by community health centers, access to professional dietary consultation, and respite care services. Interventions of this kind have been shown to reduce caregiver burnout and improve both care quality and caregiver retention in other settings, particularly when implemented within a broader system of elder care coordination (28, 29).

These proposed solutions must be embedded within local systems of care and tailored to the heterogeneity of caregivers identified in this study. Professional caregivers, family members, and volunteers differ not only in their baseline competencies but also in their motivations and access to resources (30, 31). Designing interventions that respond to these differences may enhance engagement and long-term effectiveness. For example, family caregivers may benefit from flexible learning modules, while volunteers could be integrated into structured community health teams with ongoing supervision. Ultimately, improving nutritional care for functionally disabled older adults requires a shift in how caregiving roles are supported and understood. Isolated interventions are unlikely to yield sustainable improvements unless they are situated within a more coordinated framework that includes training, supervision, emotional support, and institutional recognition of caregivers’ contributions (32, 33).

This study has several limitations that should be considered when interpreting the findings. First, the cross-sectional design precludes any inference of causality among knowledge, attitudes, and practices. Second, data were collected through self-reported online questionnaires, which may be subject to recall bias and social desirability bias. Third, the attitudinal component was operationalized using the ZBI-12, which measures caregiver burden rather than attitudes specifically related to nutritional management; this conceptual mismatch should be taken into account when interpreting the findings. Fourth, the study sample was recruited through convenience sampling in hospital settings, which may introduce selection bias. Caregivers without hospital contact, such as those providing care entirely at home, may differ in knowledge, burden, and practices. Fifth, the study was limited to a specific geographic region, which may further restrict the generalizability of the results to other populations or caregiving settings. Additionally, this study did not account for potential unmeasured variables (e.g., access to health services, social support), which may influence the SEM results. Although our sample size (*N* = 550) exceeds commonly recommended thresholds for SEM, the number of estimated parameters in the model may still affect the stability of some parameter estimates., larger samples would further improve the stability and generalizability of the parameter estimates.

In conclusion, caregivers of functionally disabled older adults demonstrated limited nutritional knowledge, accompanied by moderately negative attitudes, yet relatively active practices, suggesting a disconnect between awareness and behavioral execution in nutritional care. Targeted educational interventions that enhance caregivers’ nutritional knowledge may help foster more positive attitudes and, in turn, further improve caregiving practices in clinical and community settings. Given that greater knowledge was associated with increased caregiver burden in our study, educational programs should be designed not only to enhance nutritional knowledge but also to mitigate potential stress. For example, training sessions could integrate modules on coping strategies, stress management, and problem-solving skills alongside nutrition education. In addition, linking caregivers to community resources and peer support networks may help reduce the emotional load associated with caregiving. Tailored, interactive, and context-specific training formats, such as workshops or online programs with practical case simulations, could further improve both the accessibility and the effectiveness of interventions. By combining knowledge transfer with psychosocial support, such programs may optimize both caregiver well-being and care outcomes.

## Data Availability

The original contributions presented in the study are included in the article/Supplementary material, further inquiries can be directed to the corresponding author.

## Footnotes

^1^ https://www.wjx.cn

## References

[1] Norman K Haß U Pirlich M. Malnutrition in older adults-recent advances and remaining challenges. Nutrients. (2021) 13:2764. doi: 10.3390/nu13082764, PMID: 34444924 PMC8399049

[2] Kudo S Mutisya E Nagao M. Population aging: an emerging research agenda for sustainable development. Soc Sci. (2015) 4:940–66. doi: 10.3390/socsci4040940

[3] Nishioka S Wakabayashi H. Interaction between malnutrition and physical disability in older adults: is there a malnutrition-disability cycle? Nutr Rev. (2023) 81:191–205. doi: 10.1093/nutrit/nuac047, PMID: 35831980

[4] Cereda E Pedrolli C Klersy C Bonardi C Quarleri L Cappello S. Nutritional status in older persons according to healthcare setting: a systematic review and meta-analysis of prevalence data using MNA(®). Clin Nutr. (2016) 35:1282–90. doi: 10.1016/j.clnu.2016.03.008, PMID: 27086194

[5] Volkert D Beck AM Cederholm T Cruz-Jentoft A Goisser S Hooper L. ESPEN guideline on clinical nutrition and hydration in geriatrics. Clin Nutr. (2019) 38:10–47. doi: 10.1016/j.clnu.2018.05.024, PMID: 30005900

[6] Mj K. Frequency of malnutrition in older adults: a multinational perspective using the mini nutritional assessment. J Am Geriatr Soc. (2010) 58:1734–8. doi: 10.1111/j.1532-5415.2010.03016.x 20863332

[7] Parveen S Mehra A Kumar K Grover S. Knowledge and attitude of caregivers of people with dementia. Geriatr Gerontol Int. (2022) 22:19–25. doi: 10.1111/ggi.14304, PMID: 34755432

[8] Niu S Ding S Wu S Ma J Shi Y. Correlations between caregiver competence, burden and health-related quality of life among Chinese family caregivers of elderly adults with disabilities: a cross-sectional study using structural equations analysis. BMJ Open. (2023) 13:e067296. doi: 10.1136/bmjopen-2022-067296, PMID: 36806142 PMC9944642

[9] Sun L-l Zheng L Chen L-l Wang Z-d Li Q Liu L. Experiences of formal caregivers of elderly inpatients with physical disabilities in China: a qualitative study. BMC Nurs. (2024) 23:392. doi: 10.1186/s12912-024-02019-3, PMID: 38849821 PMC11157724

[10] Chen Z Zhou X Jiang L Song C Wang S Zhao H. Knowledge, attitudes, and practices of family caregivers for patients with cerebral infarction toward home-based care. Front Public Health. (2024) 12:1436423. doi: 10.3389/fpubh.2024.1436423, PMID: 39228843 PMC11368753

[11] Zhao W Jones C Wu ML Moyle W. Healthcare professionals' dementia knowledge and attitudes towards dementia care and family carers' perceptions of dementia care in China: an integrative review. J Clin Nurs. (2022) 31:1753–75. doi: 10.1111/jocn.15451, PMID: 32786146

[12] School of Nutrition C C o E M E, Resuscitation B K L o C C, Clinical Research Center of Emergency Medicine B C H, Capital Medical University. Recommendations for home nutrition therapy for disabled patients in China. Emerg Med China. (2021) 10:829–41. doi: 10.3969/j.issn.1002-1949.2021.10.001

[13] Association C N a T B o Z M, Association N P C o Z M D, Association P a E N B o Z M, Association N C o Z R M, Association N H I E M, Branch Z J N S a T P C o M N I . Expert consensus on nutrition diagnosis and treatment in elderly patients. Zhejiang Med J. (2023) 45:113–20. doi: 10.12056/j.issn.1006-2785.2023.45.2.2022-154

[14] Bédard M Molloy DW Squire L Dubois S Lever JA O'Donnell M. The zarit burden interview: a new short version and screening version. Gerontologist. (2001) 41:652–7. doi: 10.1093/geront/41.5.652, PMID: 11574710

[15] Aloudiny WH Alsaran FF Alessa FM Almoayad F Fiala L. Examining emotional and physical burden in informal Saudi caregivers: links to quality of life and social support. Healthcare (Basel). (2024) 12:1851. doi: 10.3390/healthcare12181851, PMID: 39337192 PMC11431032

[16] Lee F Suryohusodo AA. Knowledge, attitude, and practice assessment toward COVID-19 among communities in East Nusa Tenggara, Indonesia: a cross-sectional study. Front Public Health. (2022) 10:957630. doi: 10.3389/fpubh.2022.957630, PMID: 36388283 PMC9659730

[17] Sim M Kim SY Suh Y. Sample size requirements for simple and complex mediation models. Educ Psychol Meas. (2022) 82:76–106. doi: 10.1177/00131644211003261, PMID: 34992307 PMC8725051

[18] Shahin MAH Hussien RM. Knowledge, attitude, practice, and self-efficacy of caregivers of children with epilepsy: impact of a structured educational intervention program. Epilepsy Seizure. (2021) 13:1–16. doi: 10.3805/eands.13.1

[19] Chebib N Waldburger TC Boire S Prendki V Maniewicz S Philippe M. Oral care knowledge, attitude and practice: caregivers’ survey and observation. Gerodontology. (2021) 38:95–103. doi: 10.1111/ger.12502, PMID: 33073432

[20] Maciel MM Reiners AAO Cunha CRT Azevedo RC d S Cardoso JDC Andrade AC d S. Analysis of knowledge, attitudes, and practices of physicians and nurses regarding the experiences of family caregivers of older adults with dementia: a KAP study. Rev Bras Geriatr Gerontol. (2024) 27:e230124. doi: 10.1590/1981-22562024027.230124.pt

[21] Pedersen MRL Hansen AF. Interventions by caregivers to promote motor development in young children, the caregivers’ attitudes and benefits hereof: a scoping review. Int J Environ Res Public Health. (2022) 19:11543. doi: 10.3390/ijerph191811543, PMID: 36141815 PMC9517187

[22] GebreEyesus FA Tarekegn TT Amlak BT Shiferaw BZ Emeria MS Geleta OT. Pediatric health. Med. Ther. (2019) 12:223–38. doi: 10.2147/PHMT.S295378, PMID: 34007240 PMC8121275

[23] Jalil H Chong M-C Jalaludin MY Wong LP Hmwe NTT. Knowledge, attitude, and practice among mothers toward breastfeeding and complementary feeding in community health setting, Malaysia. Heliyon. (2024) 10:e39746. doi: 10.1016/j.heliyon.2024.e39746, PMID: 39553637 PMC11564990

[24] Momoh FE Olufela OE Adejimi AA Roberts AA Oluwole EO Ayankogbe OO. Mothers’ knowledge, attitude and home management of diarrhoea among children under five years old in Lagos, Nigeria. Afr J Prim Health Care Fam Med. (2022) 14:3119. doi: 10.4102/phcfm.v14i1.3119, PMID: 35695440 PMC9210141

[25] Nassar AA Fatani BA Almobarak OT Alotaibi SI Alhazmi RA Marghalani AA. Knowledge, attitude, and behavior of parents regarding early childhood caries prevention of preschool children in Western region of Saudi Arabia: a cross-sectional study. Dent J. (2022) 10:218. doi: 10.3390/dj10120218, PMID: 36547034 PMC9777336

[26] Temsah M-H Aljamaan F Alhaboob A Almosned B Alsebail R Temsah R. Enhancing parental knowledge of childhood and adolescence safety: an interventional educational campaign. Medicine. (2022) 101:e28649. doi: 10.1097/MD.0000000000028649, PMID: 35060555 PMC8772645

[27] Zhang M Zhang H Hu S Zhang M Fang Y Hu J. Investigation of anxiety, depression, sleep, and family function in caregivers of children with epilepsy. Front Neurol. (2021) 12:744017. doi: 10.3389/fneur.2021.744017, PMID: 34764930 PMC8575681

[28] Adum P Agyare VA Owusu-Marfo J Agyeman YN. Knowledge, attitude and practices of malaria preventive measures among mothers with children under five years in a rural setting of Ghana. Malar J. (2023) 22:268. doi: 10.1186/s12936-023-04702-3, PMID: 37700321 PMC10498521

[29] Yang J Lin L Gao Y Wang W Yuan L. Interventions and strategies to improve social support for caregivers of children with chronic diseases: an umbrella review. Front Psych. (2022) 13:973012. doi: 10.3389/fpsyt.2022.973012, PMID: 36213907 PMC9537372

[30] Saaka M Wemah K Kizito F Hoeschle-Zeledon I. Effect of nutrition behaviour change communication delivered through radio on mothers’ nutritional knowledge, child feeding practices and growth. J Nutr Sci. (2021) 10:e44. doi: 10.1017/jns.2021.35, PMID: 34164123 PMC8190717

[31] Vicens-Blanes F Miró-Bonet R Molina-Mula J. Analysis of the perceptions, knowledge and attitudes of parents towards fever in children: a systematic review with a qualitative meta-synthesis. J Clin Nurs. (2023) 32:969–95. doi: 10.1111/jocn.16271, PMID: 35224809

[32] Almutairi WM Alsharif F Khamis F Sallam LA Sharif L Alsufyani A. Assessment of mothers’ knowledge, attitudes, and practices regarding childhood vaccination during the first five years of life in Saudi Arabia. Nurs Rep. (2021) 11:506–16. doi: 10.3390/nursrep11030047, PMID: 34968325 PMC8608048

[33] Kwerengwe RI Singh K. Impact of knowledge, attitude and practice of mothers regarding complementary feeding on the nutritional status of children. SALT J Sci Res Healthc. (2023) 3:01–12. doi: 10.56735/saltjsrh.ms2303020112

