# Supplementary material for: Knowledge, attitudes, and practices of caregivers of functionally disabled older adults regarding nutritional management
Source: Front Nutr. 2025 Nov 5;12:1660965. doi: 10.3389/fnut.2025.1660965 (PMC12627031; doi:10.3389/fnut.2025.1660965)
Supplement: Supplementary file 1 [file Table_1.DOCX]

**Table S1. Model fitting indices of Confirmatory Factor Analysis (CFA)**

| Indicators | Reference | Actual |
| --- | --- | --- |
| CMIN/DF | 1-3: Excellent, 3-5: Good | 3.564 |
| RMSEA | <0.08: Good | 0.068 |
| IFI | >0.8: Good | 0.845 |
| TLI | >0.8: Good | 0.833 |
| CFI | >0.8: Good | 0.844 |

**Table S2. List of participating hospitals in this study**

| **No.** | **Province** | **Hospital Name** | **Department** |
| --- | --- | --- | --- |
| 1 | Guangdong | Xuwen County Second People's Hospital, Zhanjiang | Department of Respiratory and Critical Care |
| 2 | Guangdong | Guangdong Work Injury Rehabilitation Hospital | Department of Rehabilitation |
| 3 | Guangdong | Guangdong Second People's Hospital | Department of Rehabilitation |
| 4 | Guangdong | Guangzhou Qinzheng Traditional Chinese Medicine Hospital | Department of Rehabilitation |
| 5 | Guangdong | Shenzhen First People's Hospital | Department of Rehabilitation |
| 6 | Guangdong | Guangzhou Dongsheng Hospital | Department of Rehabilitation |
| 7 | Guangdong | Guangdong Hospital of Integrated Traditional Chinese and Western Medicine | Department of Cardiology |
| 8 | Guangdong | Huizhou First People's Hospital | Department of Rehabilitation |
| 9 | Guangdong | Zhongluotan Town Health Center | Department of Cardiology |
| 10 | Guangxi | Guangxi Zhuang Autonomous Region National Hospital | Department of Rehabilitation |
| 11 | Guangxi | Guangxi University of Chinese Medicine First Affiliated Hospital | Department of Rehabilitation |
| 12 | Guangxi | Fangchenggang First People's Hospital | Department of Neurology |
| 13 | Hunan | Yiyang Third People's Hospital | Department of Rehabilitation |

**Table S3. Post-hoc test of demographic data**

|  | | Knowledge | | | Attitude | | | Practice | | |
| --- | --- | --- | --- | --- | --- | --- | --- | --- | --- | --- |
|  |  | Statistic | P | Bonferroni P | Statistic | P | Bonferroni P | Statistic | P | Bonferroni P |
| Household monthly income (Yuan) | <5000 VS 5000-10000 | 1.149 | 0.250 | 0.999 | -1.983 | 0.047 | 0.284 | 0.880 | 0.379 | 0.999 |
|  | <5000 VS 10000-20000 | 3.541 | <0.001 | 0.002 | -7.234 | <0.001 | <0.001 | 3.444 | <0.001 | 0.003 |
|  | <5000 VS >20000 | 4.478 | <0.001 | <0.001 | -1.591 | 0.112 | 0.669 | 3.962 | <0.001 | <0.001 |
|  | 5000-10000 VS 10000-20000 | 2.633 | 0.008 | 0.051 | -5.680 | <0.001 | <0.001 | 2.757 | 0.006 | 0.035 |
|  | 5000-10000 VS 10000-20000 | 3.909 | <0.001 | 0.001 | -0.583 | 0.560 | 0.999 | 3.529 | <0.001 | 0.003 |
|  | 10000-20000 VS >20000 | 2.141 | 0.032 | 0.194 | 2.762 | 0.006 | 0.034 | 1.711 | 0.087 | 0.523 |
| Relationship with the functionally disabled older person | Immediate family member VS Non-immediate family member | -1.681 | 0.093 | 0.557 | 1.501 | 0.133 | 0.800 | -1.789 | 0.074 | 0.442 |
|  | Immediate family member VS Professional caregiver | -6.412 | <0.001 | <0.001 | 4.243 | <0.001 | <0.001 | -3.931 | <0.001 | 0.001 |
|  | Immediate family member VS Volunteer or staff from other rehabilitation institutions | -2.137 | 0.033 | 0.196 | 2.396 | 0.017 | 0.099 | -1.471 | 0.141 | 0.849 |
|  | Non-immediate family member VS Professional caregiver | -2.347 | 0.019 | 0.114 | 1.173 | 0.241 | 0.999 | -0.698 | 0.485 | 0.999 |
|  | Non-immediate family member VS Volunteer or staff from other rehabilitation institutions | -0.503 | 0.615 | 0.999 | 0.845 | 0.398 | 0.999 | 0.116 | 0.908 | 0.999 |
|  | Professional caregiver VS Volunteer or staff from other rehabilitation institutions | 1.517 | 0.129 | 0.776 | -0.040 | 0.968 | 0.999 | 0.773 | 0.440 | 0.999 |
| Health condition of the functionally disabled older person | Excellent VS Good | -1.761 | 0.078 | 0.782 | 0.592 | 0.554 | 0.999 | -1.815 | 0.070 | 0.696 |
|  | Excellent VS Fair | -1.402 | 0.161 | 0.999 | -1.350 | 0.177 | 0.999 | -0.987 | 0.324 | 0.999 |
|  | Excellent VS Poor | -0.509 | 0.611 | 0.999 | -3.280 | 0.001 | 0.010 | 0.936 | 0.349 | 0.999 |
|  | Excellent VS Very poor | 1.417 | 0.156 | 0.999 | -2.741 | 0.006 | 0.061 | 1.233 | 0.218 | 0.999 |
|  | Good VS Fair | 0.754 | 0.451 | 0.999 | 0.592 | 0.554 | 0.999 | 1.285 | 0.199 | 0.999 |
|  | Good VS Poor | 1.400 | 0.162 | 0.999 | -4.197 | <0.001 | <0.001 | 3.019 | 0.003 | 0.025 |
|  | Good VS Very poor | 2.982 | 0.003 | 0.029 | -3.376 | <0.001 | 0.007 | 2.834 | 0.005 | 0.046 |
|  | Fair VS Poor | 0.946 | 0.344 | 0.999 | -2.820 | 0.005 | 0.048 | 2.396 | 0.017 | 0.166 |
|  | Fair VS Very poor | 2.797 | 0.005 | 0.052 | -2.133 | 0.033 | 0.330 | 2.245 | 0.025 | 0.248 |
|  | Poor VS Very poor | 1.941 | 0.052 | 0.523 | -0.154 | 0.878 | 0.999 | 0.524 | 0.600 | 0.999 |
| Disability level | Mild disability VS Moderate disability | 0.772 | 0.440 | 0.999 | -2.971 | 0.003 | 0.009 |  |  |  |
|  | Mild disability VS Severe disability | -2.063 | 0.039 | 0.117 | -2.057 | 0.040 | 0.119 |  |  |  |
|  | Moderate disability VS Severe disability | -3.090 | 0.002 | 0.006 | 0.981 | 0.327 | 0.980 |  |  |  |

**Table S4. Correlation analysis among KAP**

|  | Knowledge | Attitude | Practice |
| --- | --- | --- | --- |
| Knowledge | 1 |  |  |
| Attitude | -0.391 (P<0.001) | 1 |  |
| Practice | 0.642 (P<0.001) | -0.476 (P<0.001) | 1 |

**Table S5. Model fitting of SEM**

| **Fit index** | **Ref.** | **Model index** |
| --- | --- | --- |
| **CMIN/DF** | 1-3 excellent，3-5 good | 3.564 |
| **RMSEA** | <0.08 good | 0.068 |
| **IFI** | >0.8 good | 0.845 |
| **TLI** | >0.8 good | 0.833 |
| **CFI** | >0.8 good | 0.844 |
