# Supplementary figures and images for: Knowledge, attitudes, and practices of caregivers of functionally disabled older adults regarding nutritional management
Source: Front Nutr. 2025 Nov 5;12:1660965. doi: 10.3389/fnut.2025.1660965 (PMC12627031; doi:10.3389/fnut.2025.1660965)

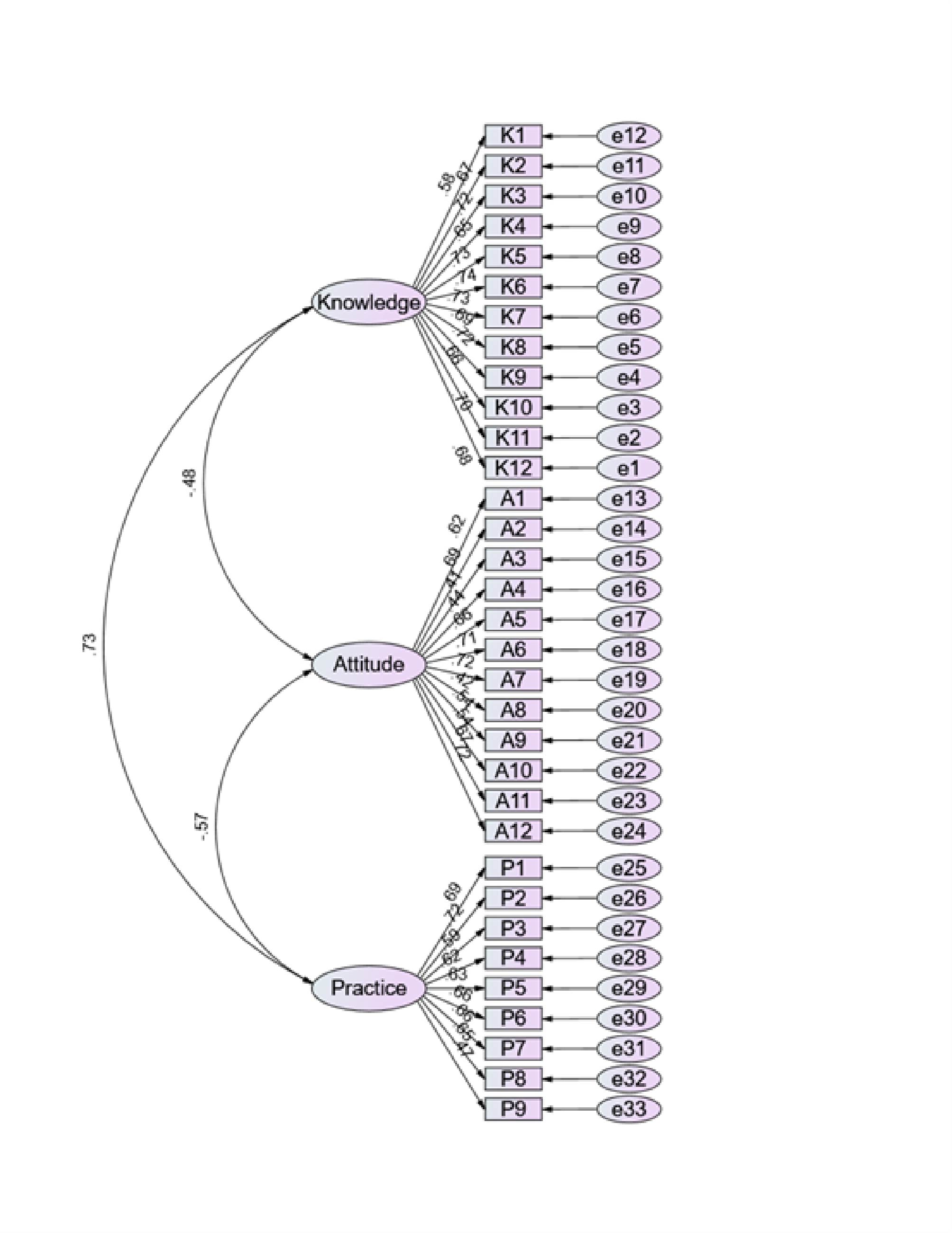

Supplement: Supplementary file 2 [file Image_1.JPEG]

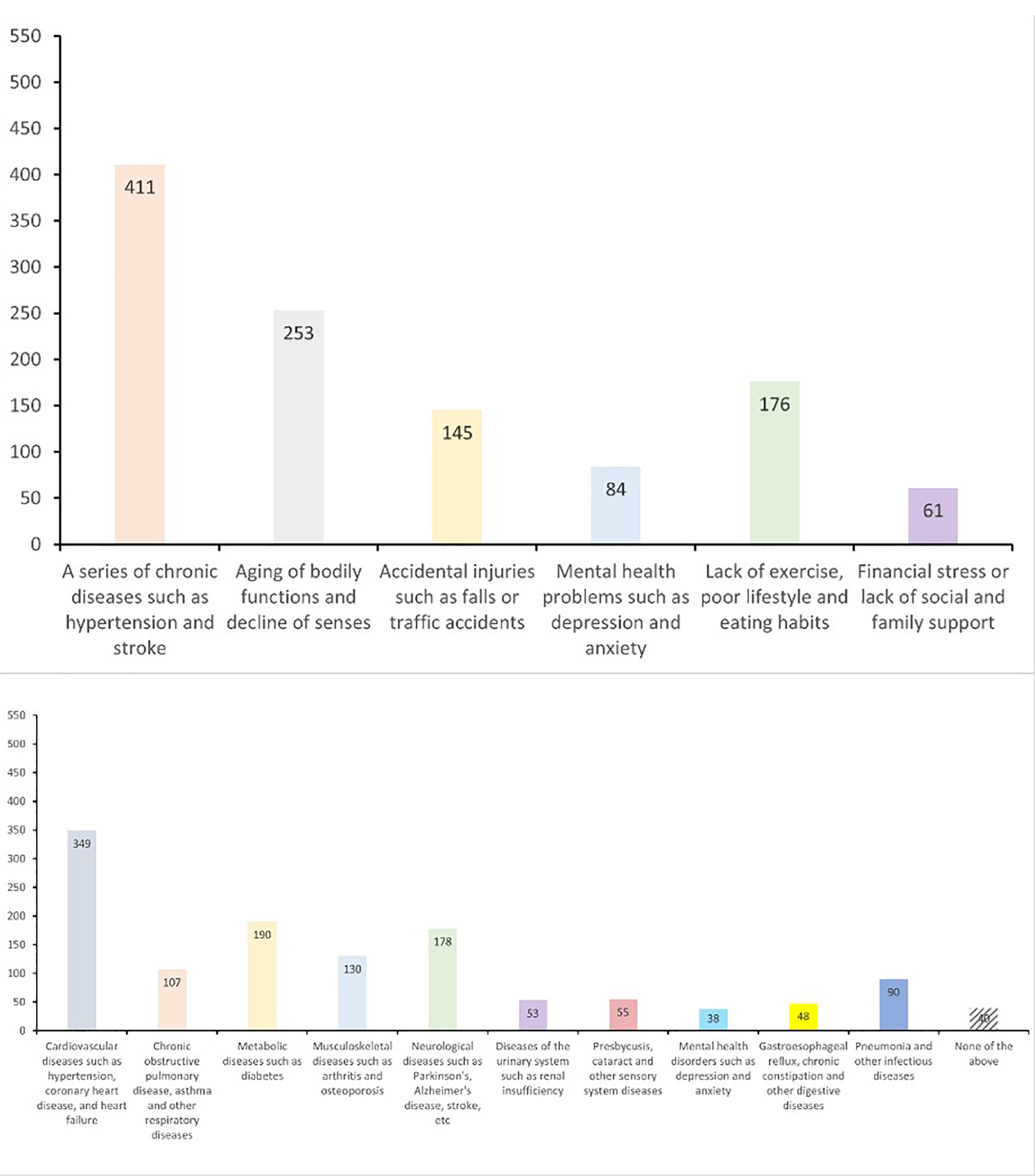

Supplement: Supplementary file 3 [file Image_2.JPEG]

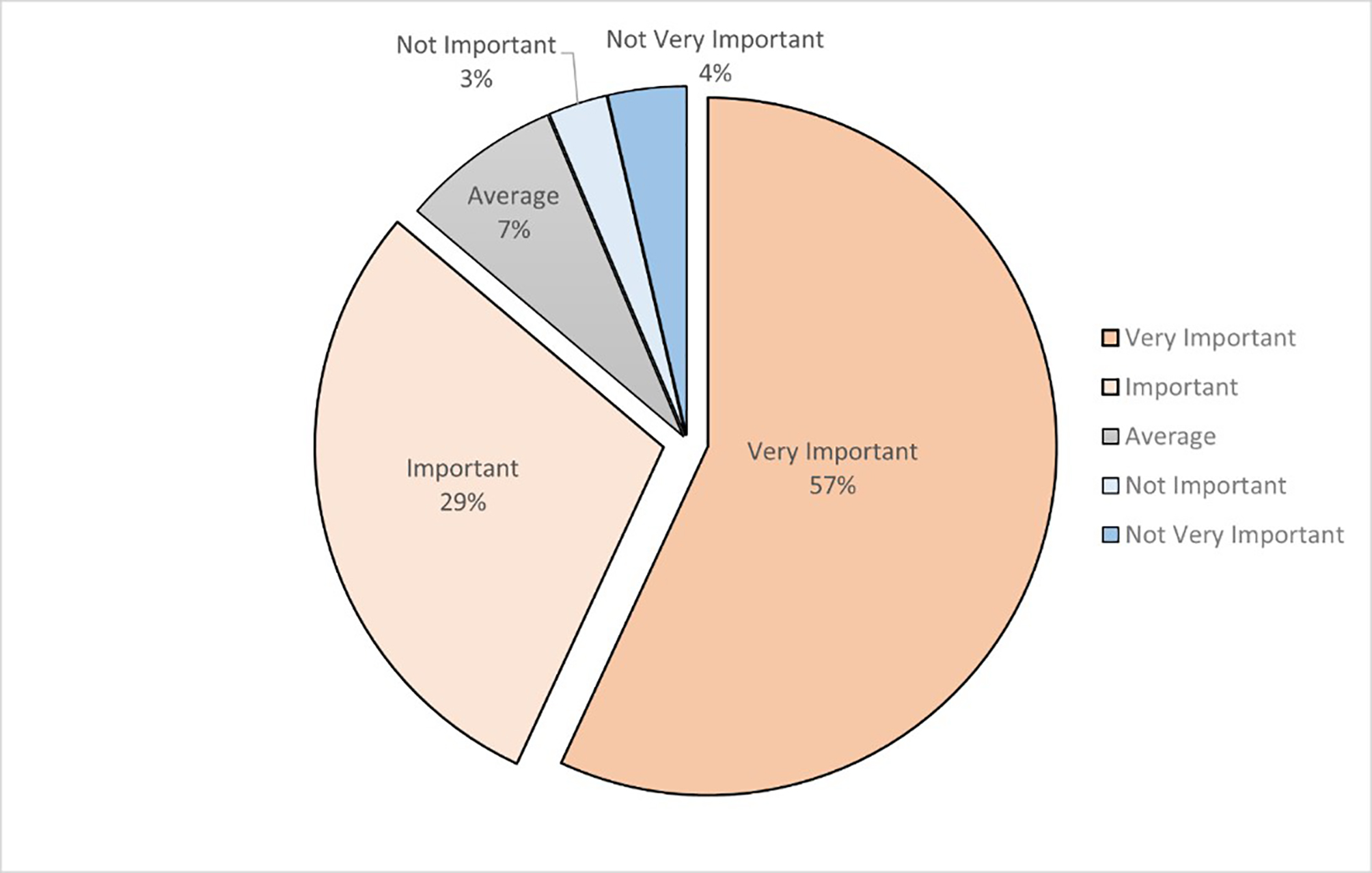

Supplement: Supplementary file 4 [file Image_3.JPEG]
